# SINEs as Credible Signs to Prove Common Ancestry in the Tree of Life: A Brief Review of Pioneering Case Studies in Retroposon Systematics

**DOI:** 10.3390/genes13060989

**Published:** 2022-05-31

**Authors:** Masato Nikaido, Hidenori Nishihara, Norihiro Okada

**Affiliations:** 1 School of Life Science and Technology, Tokyo Institute of Technology, Meguro-ku, Tokyo 152-8550, Japan; mnikaido@bio.titech.ac.jp (M.N.); hnishiha@bio.titech.ac.jp (H.N.); 2 School of Pharmacy, Kitasato University, Kanagawa 252-0373, Japan

**Keywords:** SINEs/LINEs, molecular systematics, incomplete lineage sorting

## Abstract

Currently, the insertions of SINEs (and other retrotransposed elements) are regarded as one of the most reliable synapomorphies in molecular systematics. The methodological mainstream of molecular systematics is the calculation of nucleotide (or amino acid) sequence divergences under a suitable substitution model. In contrast, SINE insertion analysis does not require any complex model because SINE insertions are unidirectional and irreversible. This straightforward methodology was named the “SINE method,” which resolved various taxonomic issues that could not be settled by sequence comparison alone. The SINE method has challenged several traditional hypotheses proposed based on the fossil record and anatomy, prompting constructive discussions in the Evo/Devo era. Here, we review our pioneering SINE studies on salmon, cichlids, cetaceans, Afrotherian mammals, and birds. We emphasize the power of the SINE method in detecting incomplete lineage sorting by tracing the genealogy of specific genomic loci with minimal noise. Finally, in the context of the whole-genome era, we discuss how the SINE method can be applied to further our understanding of the tree of life.

## 1. Introduction

Short interspersed elements (SINEs) are a class of mobile repetitive sequences called retroposons that range from 70 to 500 bp in length and are present at more than 100,000 copies in mammalian genomes. SINEs are amplified by retroposition through a “copy–paste” mechanism, in which a SINE is transcribed to produce an RNA that is then reverse-transcribed to produce a cDNA, which is subsequently integrated at another genomic locus [1]. SINE retroposition is achieved with the assistance of proteins encoded by a partner long interspersed element (LINE) [2,3], which is another type of retroposon approximately 6000 bp in length [4]. Thus, SINEs are distinct from simple tandem repeats, which are amplified by gene duplication at the DNA level [5]. SINEs were initially identified in the human genome as 300-nucleotide repeated sequences called the *Alu* family [6], which were derived from 7SL RNA [7]. However, our research group found that most SINE sequences in mammals represented by rats, rabbits, and cattle are derived from tRNAs [8], and this was confirmed by SINEs of galago [9]. Subsequently, SINEs were also discovered in diverse animal taxa: mollusks such as squid [10] and octopus [11,12], fishes such as salmon [13] and cichlids [14], newts [11], and even plants [15]; all such SINEs were found to be derived from tRNAs, thus providing a comprehensive understanding of the origin and taxonomic distribution of SINEs [1,5].

The “copy–paste” mechanism of retroposition is distinct from the “cut–paste” mechanism of transposition. The copy–paste of SINEs and LINEs implies that their insertion into the genome is irreversible, which is crucial for the accuracy of molecular phylogenetic inference. In molecular phylogenetics, nucleotide (or amino acid) substitutions have conventionally been used to calculate the relative divergence of gene sequences between species. However, the possible occurrence of back/repeated mutations necessitates the use of suitable substitution models for divergence calculations. However, the insertion of SINEs is unidirectional and, therefore, analyses of SINEs do not require complex substitution models but rather require only a simple, parsimonious model to elucidate the common ancestry. Namely, species sharing SINE insertions at orthologous loci can be treated as monophyletic. This simple yet powerful SINE insertion analysis was named the "SINE method" and has been used often to construct phylogenetic trees [1,16]. In principle, LINEs also can be used for phylogenetic inference, especially for resolving the deep branching of mammals [17,18,19], but SINEs are most often used because their lengths are amenable to PCR-based detection of insertions. Details of the relevant experimental procedures are described in our previous reviews [1,20].

The pioneering work of the SINE method involved elucidating the phylogenetics of salmonids and cichlids, which revealed the monophyly of several taxonomic clades, demonstrating the validity of the SINE method for phylogenetic inferences [21,22]. Subsequently, much attention was paid to the success in phylogenetic elucidation of cetaceans and even-toed ungulates, which clarified the origin of whales and settled problems concerning the paraphyly or monophyly of toothed whales [23,24,25]. The SINE method has also been applied to infer relationships among other mammals, such as primates [26,27,28], Afrotherians [29], bats [30], rodents [31], marsupials [32,33], and turtles [34,35]. Until recently, the insertion of SINEs at particular loci has been detected by screening genomic libraries with subsequent flanking PCR, the so-called wet experiments [20]. However, with the increase in the number of whole-genome sequences registered in databases, bioinformatic techniques have been used to detect both SINE and LINE insertions for phylogenetic inference, as represented by our recent work on waterbird phylogeny [36,37]. As our phylogenetic studies became extended to encompass a broad range of animal groups, we discovered examples of inconsistent patterns of SINE insertions among loci of salmonids and cichlids. When the inconsistencies were initially observed, the validity of the SINE method for phylogenetic inference was once questioned. However, we later found that such inconsistencies were caused by incomplete lineage sorting (ILS), in which unfixed polymorphisms were allocated to descendant species owing to rapid divergence [38]. Thus, the accuracy of the SINE method for determining the genealogy of each locus became firmly established [38,39]. Examples of ILS were further detected among baleen whales [40], Laurasiatherian mammals [17,19], and three major mammalian clades [31], implying that these groups underwent rapid divergence. Our research group also discovered that SINE sequences themselves have functions, e.g., as enhancers and in exonization. In this review, however, we focus on the aforementioned molecular phylogenetic studies based on results of the SINE method conducted by our group.

## 2. Actual Outcomes of the SINE Method

### 2.1. Salmonid Phylogeny as a Pioneering Study of the SINE Method

**Figure 1:**
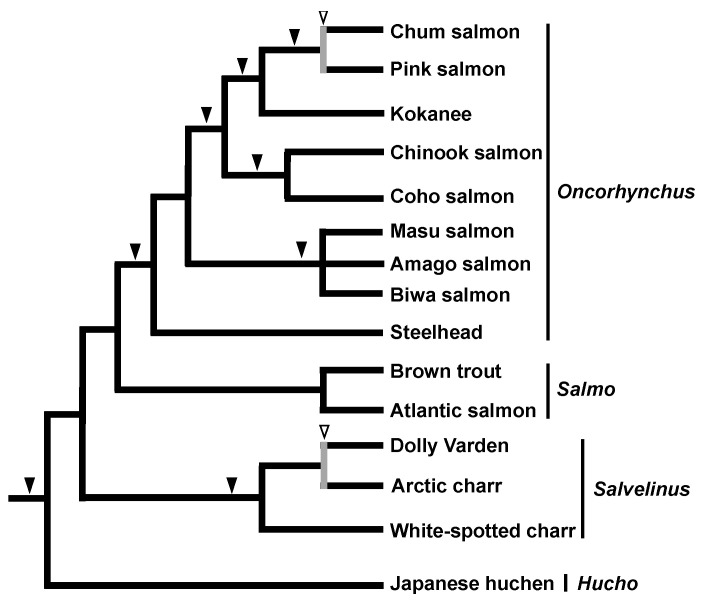
Phylogenetic tree of salmonid fishes based on results of the SINE method. Black arrowheads indicate SINE insertions with no inconsistency among loci. White arrowheads with gray vertical bars indicate the detection of an inconsistent SINE insertion among loci, which is attributable to incomplete lineage sorting, as detected in Chum and Pink salmon [46] as well as Dolly Varden and Arctic charr [47].

The pioneering contributions to SINE phylogenetics were achieved by a series of studies on salmonids. Our group discovered three SINE families derived from tRNA^Lys^ in the salmon genome (Sma I, Fok I, and Hpa I) [13,41]. We then used these SINEs to elucidate the phylogenetic relationships among salmonid fish. At that time, it was already known that the Alu family consists of several subfamilies that can be distinguished by diagnostic nucleotide changes. A comparison of these sequences with 7SL revealed that these Alu subfamilies have been replaced during the evolution of primates [42]. Similarly, in salmonids, SINE families and subfamilies are distributed to specific clades, implying that they have been replaced during evolution [41,43]. Specifically, Sma I is distributed exclusively in pink and chum salmon and Fok I is distributed in a group of salvelinus species, whereas Hpa I is widely distributed among salmonid species. This implies that the distribution of SINE families and/or subfamilies may reflect the phylogeny of salmonids. However, our research group also came up with a new idea, that the presence or absence of a SINE insertion at a particular locus may reflect the phylogeny of a particular group, i.e., the sharing of a common ancestor, and hence we conducted a practical study of this idea [21]. The results demonstrated the monophyly of Pacific salmons (Genus: *Oncorhynchus*) by the insertion of a SINE into the Hpa-345 locus. We also showed that the steelhead trout, for which the phylogenetic position was debatable at the time, is a distinct group from other trout. In addition, several internal phylogenies of Pacific salmon [22] as well as the monophyly of masu salmon [44] were revealed by the SINE method, demonstrating the power of this approach for applications in molecular systematics (Figure 1). Further vigorous characterization of SINE loci led to the identification of a number of species-specific SINE insertions, revealing that the rate of SINE amplification varies greatly among species [45]. In particular, the presence of unfixed SINE insertions in Chum salmon [46] and Salvelinus [47] has been a cornerstone of our understanding of ancestral polymorphisms and ILS (see later section).

### 2.2. Cichlid Phylogeny

**Figure 2:**
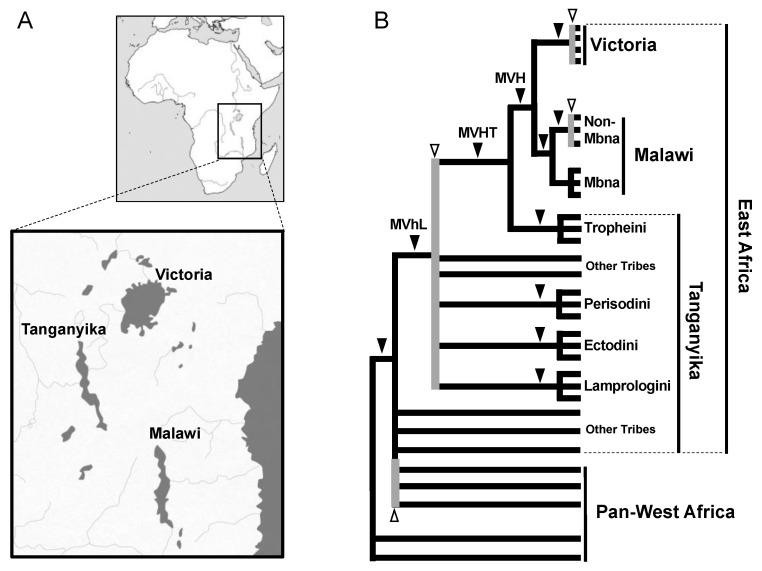
Phylogeny of East African cichlids. (**A**) Geography of the East African Great Lakes Victoria, Tanganyika, and Malawi. (**B**) Phylogenetic tree of East African cichlids based on results of the SINE method. Most major clades, such as Lake Victoria (V), Lake Malawi (M), MVH (M + V + Haplochromini), MVHT (MVH + Tropheini), MVhL (MVHT + Eretmodini, Perissodini, Cyprichromini, Limnochromini, Ectodini, and Lamplologini), and each of the four tribes in Tanganyika, are supported by multiple consistent SINE insertions (black arrowheads). SINE insertion patterns are inconsistent among the species in Lake Victoria, among non-Mbna species in Lake Malawi, and among the tribes of Lake Tanganyika, demonstrating that ILS was caused by rapid speciation (white arrowheads with gray vertical lines). Note that inconsistent SINE insertions were also detected in non-East Africa lineages, implying that hidden radiation occurred before the radiation of East African cichlids [56].

We next adopted the SINE method to infer the phylogeny of cichlids inhabiting the three East African Great Lakes (Tanganyika, Malawi, and Victoria). These fish have attracted the attention of many researchers as textbook examples of adaptive evolution because each lake has several hundred endemic species [48]. Phylogenetic analyses based on sequences of the mitochondrial control region [49] and allozyme data at 21 loci [50] have suggested that the cichlids of Lake Victoria and Lake Malawi each form a monophyletic group and that Lake Tanganyika may be the evolutionary reservoir of the East African cichlids. However, these analyses included only a few groups among many cichlid species, and therefore a detailed assessment was not carried out. Our group discovered the AFC family of tRNA-derived SINEs, which are specifically distributed in the genomes of East African cichlids [14], as well as a partner LINE (CiLINE2) that shares a 3’ end sequence [51]. Based on SINE insertions, we first demonstrated the monophyly of the four major tribes of Lake Tanganyika cichlids, namely Lamprologini, Ectodini, Tropheini, and Perissodini [14]. Later studies characterized SINE insertions that suggested the monophyly of cichlids in each of Lake Malawi [52] and Lake Victoria [53] as well as the monophyly in Lake Victoria, Malawi cichlids and the Tropheini tribe (Figure 2) [53,54]. These studies demonstrated that the SINE method is applicable to various fish species. However, as we intensively worked on the SINE analysis of cichlids, we observed intraspecific ancestral polymorphisms and inconsistent loci that were originally documented in salmon. For example, SINE insertion patterns among cichlids that radiated from Lakes Victoria and Malawi were not fixed within species [52,53], which is consistent with the existence of neutral SNPs that are not fixed but shared among species [55]. Furthermore, we found that SINE insertion patterns were not consistent among loci of the tribes of Lake Tanganyika, suggesting that an ancestral polymorphism during adaptive radiation became randomly fixed in each lineage after divergence. This is a clear example of how SINE insertion analysis can detect ILS (Figure 2) [54].

### 2.3. The Origin of Cetaceans

**Figure 3:**
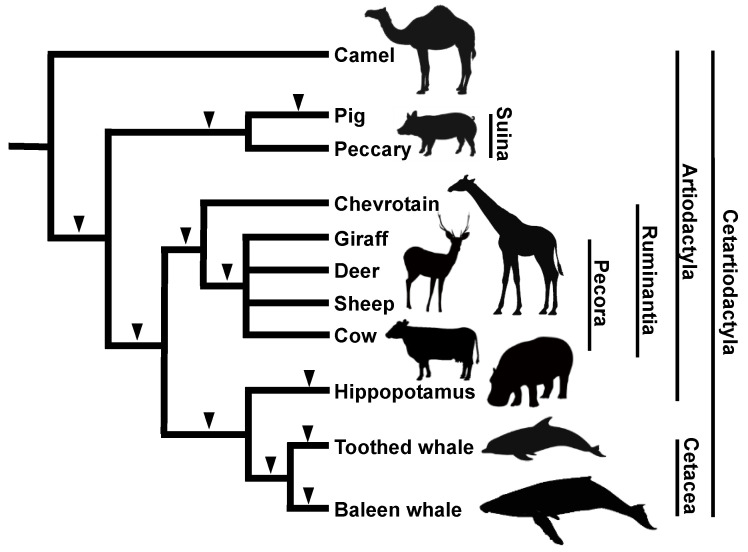
Phylogenetic tree of cetaceans and artiodactyls based on results of the SINE method. The insertion of SINEs clearly revealed the branching order of these groups (black arrowheads). The monophyly of cetaceans and hippopotamuses was revealed by several independent SINE insertions. Note that the artiodactyls are not monophyletic, which led to the designation of the new classification called Cetartiodactyla. No inconsistent SINE insertion patterns were detected during the analysis of this group.

One of the most noteworthy discoveries by our group using the SINE method is the origin of cetaceans. According to paleontological and morphological studies, it was becoming increasingly clear that the order Cetacea (whales and dolphins) is more closely related to the order Artiodactyla (even-toed ungulates) than to other ungulates [57,58]. Furthermore, molecular phylogenetic analysis using mitochondrial gene sequences led to the striking hypothesis that the order Cetacea is included within the order Artiodactyla [59]. Although the statistical fragility of that study was pointed out [60], additional molecular phylogenetic studies suggested the inclusion of cetaceans within Artiodactyla. Our group characterized two novel tRNA^Glu^-derived SINE families, called CHR-1 (identified in Cetacea, Hippopotamidae, and Ruminantia) and CHR-2 [61]. We used these SINEs to resolve the Cetacea–Artiodactyla issue and clearly demonstrate that three SINE loci are shared among cetaceans, ruminants, and hippopotamuses but not among camels and pigs, thus strongly suggesting the paraphyly of the order Artiodactyla (Figure 3) [23]. However, the closest relative to cetaceans remained debatable. Actually, certain molecular phylogenetic studies based on nuclear DNA sequence comparisons suggested a close relationship between cetaceans and hippopotamuses [62,63] but had yet to be confirmed by different approaches. We addressed this issue using the SINE method and identified four independent SINE loci shared by hippopotamuses and cetaceans with no inconsistencies, strongly suggesting the monophyly of these groups [25]. We revealed the divergence order of cetaceans and artiodactyls as follows: first, Tylopoda (camels and llamas); second, Suiformes (pigs and peccaries); third, Ruminantia (chevrotains and pecorans, such as cows, deer, and giraffes); and finally, hippopotamuses and cetaceans (Figure 3). The results attained with the SINE method challenged traditional hypotheses in taxonomy as well as paleontology [64]. First, considering that all the extant members of the order Cetacea are included in the order Artiodactyla, the taxonomic name of the two orders was revised to the novel order “Cetartiodactyla.” Second, an even more controversial issue was the reconsideration of the prevailing hypothesis in paleontology regarding the phylogenetic position of mesonychids. In paleontology, the extinct mesonychids were long believed to be closely related to cetaceans but not to even-toed ungulates. Therefore, the results of the SINE method raised the two alternate possibilities that either mesonychids are nested within the artiodactyls or that mesonychids are not closely related to cetaceans. The key to solving this problem was the double-pulley structure within the astragalus. Paleontologists found that the double-pulley structure, which is characteristic of even-toed ungulates, existed in the hind limbs of the extinct cetacean, pakicetus, but not in mesonychids, suggesting that cetaceans are closely related to artiodactyls but not to mesonychids [65]. Subsequent discoveries in paleontology eventually supported our SINE tree [66], and Cetartiodactyla has been adopted as a well-established taxonomic name.

### 2.4. The Monophyly or Paraphyly of Toothed Whales

**Figure 4:**
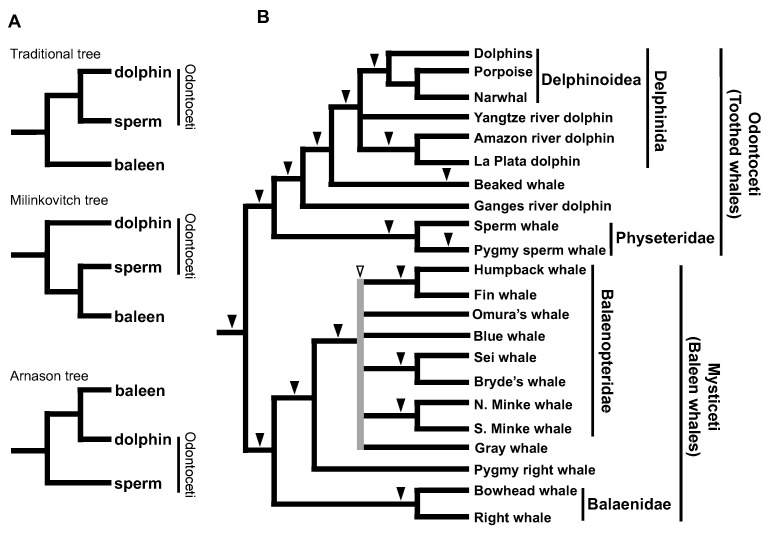
Phylogenetic trees to address the monophyly or paraphyly of toothed whales. (**A**) Three alternative hypotheses for cetacean trees. Although the conventional morphology-based hypothesis supports the monophyly of Odontoceti (toothed whales), both the Milinkovitch and Arnason hypotheses support the paraphyly of Odontoceti. (**B**) Phylogenetic tree of cetaceans based on results of the SINE method, clearly supporting the monophyly of Odontoceti. The paraphyly of river dolphins is also demonstrated, with no inconsistency in SINE insertion patterns. However, we detected inconsistent SINE loci during our analysis of the genomes of balaenopterids (white arrowheads), implying that rapid speciation occurred for this family [40,75]. For minke whales, “N.” and “S.” indicate North Atlantic and Antarctic, respectively.

In the 1990s, many of the traditional hypotheses of taxonomy based on paleontology were being revised as a result of molecular systematics studies and the power of phylogenetic reconstruction by sequence comparison was being emphasized. However, the issue of toothed whale monophyly or paraphyly highlighted one of the potential pitfalls of molecular phylogeny and provided important insights for subsequent discussions. Traditionally, the extant cetaceans were classified into two suborders, namely Odontoceti (echolocating toothed whales) and Mysticeti (filter-feeding baleen whales), based on morphological and physiological characters [67,68]. Although this classification was believed for a long time, several phylogenetic studies based on comparisons of mitochondrial genes have questioned it. For example, an analysis of mitochondrial cytochrome b sequences supported the paraphyly of toothed whales, in which sperm whales are more closely related to baleen whales than to other toothed whales [69]. Another study also supported the paraphyly of toothed whales, in which sperm whales are positioned at the base of all cetaceans [70]. A detailed statistical analysis concluded that the phylogenetic position of sperm whales varies depending on the outgroup used in the analysis and that more species and genes must be included in the analysis [71]. Indeed, phylogenetic analyses using many gene sequences and species have been conducted since then, but it has remained difficult to conclusively address this issue with sufficient bootstrap values [72]. To solve this problem, we used the SINE method to reconstruct a phylogenetic tree of 14 cetacean species, including baleen whales, sperm whales, beaked whales, dolphins, and river dolphins. The results demonstrated the monophyly of toothed whales based on three SINE loci with no inconsistency [25]. In addition to the monophyly of toothed whales, we demonstrated the paraphyly of river dolphins in that Ganges River dolphins are separate from other river dolphins, e.g., of the Amazon, La Plata, and Yangtze Rivers (Figure 4). The monophyly of toothed whales is also supported by the distribution of a specific subfamily of CHR-2 SINE (CDO subfamily) [73] and nine other SINE loci [74]. Therefore, even with highly objective molecular phylogenetics data, certain groups exist for which phylogenetic relationships are difficult to verify by sequence comparison alone, and in such cases, the SINE method has proved effective.

### 2.5. Afrotherian Phylogeny: Integration of Informatics Techniques

**Figure 5:**
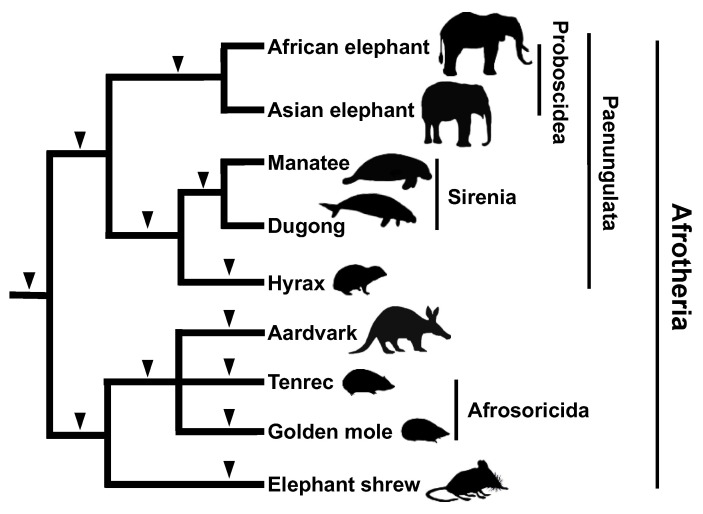
Phylogenetic tree of Afrotherian mammals based on results of the SINE method. The monophyly of each of Proboscidea and Sirenia was confirmed by several SINE insertions (black arrowheads). Note that the monophyly of each of Sirenia (manatee and dugong) and hyrax is supported by one SINE insertion. No SINE insertions were shared between Proboscidea and Sirenia. The monophyly of Afrosoricida and aardvark, which is supported by results of the SINE method, has not been suggested by sequence comparisons. To date, no contradictory SINE insertions have been detected.

Although the SINE method has been well credited after its success in the inference of cetacean phylogeny, the “wet” experiments faced technical difficulties with respect to elucidating the internal phylogeny of Afrotherian mammals. A molecular phylogenetic analysis that integrated multiple nuclear gene sequences revealed that mammals that are widely distributed in Africa (elephants, sirenians, hyraxes, aardvarks, tenrecs, golden moles, and elephant shrews) form a monophyletic group, which came to be called the Superorder “Afrotheria” [76]. However, the monophyly of Afrotheria was not accepted by most of the paleontological and anatomical studies [77]. Furthermore, the internal relationships within Afrotheria depended on which genes were used for a given analysis. We identified a novel SINE, named AfroSINE, from the genomes of Afrotherian mammals [78] and used it to elucidate phylogeny. However, because the divergence of Afrotheria dates back to 80 million years ago, their sequences have become highly differentiated, making it difficult to design PCR primers that would amplify the orthologous SINE loci for all Afrotherian species. To overcome this challenge, we conducted an inter-exon PCR [29], which is based on genome informatics and uses the human genome sequence as a reference. Specifically, primers were designed to target evolutionarily conserved exon sequences and their intervening introns (<2 kbp). PCR amplification and sequencing were then used to identify “by chance insertions” of retroposons, namely SINEs as well as LINEs. These retroposons were then used for phylogenetic inferences. The results confirmed the results of previous molecular systematics studies that supported the monophyly of Afrotheria and Paenungulata (elephants, sirenians, and hyraxes) [76], with four and five retroposon insertions, respectively. We also characterized a retroposon insertion that supported the monophyly of aardvarks and African insectivores (tenrecs and golden moles) and a sister relationship with the elephant shrew, both of which were not evident based on previous molecular phylogenetic analyses (Figure 5). Thus, the combination of bioinformatics techniques and the SINE method has overcome the major challenges of elucidating the deeper branches of the phylogenetic tree.

## 3. SINEs for Detecting Ancestral Polymorphisms and ILS

Phylogenetic inference based on the SINE method has encountered the problem of inconsistent patterns of insertions among genomic loci. Although it has been argued that such inconsistencies may violate the assumption that SINE insertions are irreversible and do not occur in the same genomic locus, it is now known that this is not the case [38]. Elucidation of the underlying cause of this phenomenon will not only validate the SINE method for phylogenetic inference but also play an important role in providing more detailed insights into the processes of speciation and population differentiation at the genome level.

### 3.1. Inconsistent SINE Loci in Salmon, Cichlids, and Baleen Whales

Inconsistency among SINE loci was first discovered in chum salmon as a polymorphism of *Sma*I SINE insertions (Figure 1) [46]. In this case, the insertion of *Sma*I SINEs before and/or after the species divergence was considered to be retained as polymorphisms. In addition, we found that polymorphisms of *Fok*I SINE insertions were shared even across two char species (Figure 1) [47]. This phenomenon was explained as follows: a SINE that becomes inserted before the divergence of two species does not remain fixed in the descendant species because of the short period of time after the divergence. A similar phenomenon has been observed in East African cichlids. For example, in Lake Victoria cichlids, which underwent adaptive radiation during a short period, the polymorphisms of AFC SINE insertions are shared within and among species [53]. The SINE polymorphisms are also shared among the non-Mbna group of Lake Malawi cichlids [52]. In the aforementioned cases, ancestral polymorphisms were detected as inconsistent SINE loci owing to the short period of time after the SINEs were inserted. However, although SINE insertions are fixed within each tribe of Lake Tanganyika cichlids [14], inconsistent SINE insertion patterns have been observed among tribes [54]. To address this issue, we proposed that SINE insertions were polymorphic in the ancestral population(s) prior to adaptive radiation in Lake Tanganyika, followed by random presence/absence fixation in each daughter lineage, which is the so-called ILS (Figure 2). Notably, inconsistent SINE loci do not represent “noise” with regard to phylogenetic inference. In other words, the detection of ILS does not indicate that the SINE method has low resolution for phylogenetic inference but rather that it is able to accurately trace the genealogy of each locus. Thus, the discovery of inconsistent SINE loci has provided strong evidence that the animal group in question has undergone rapid speciation. Our research group also detected ILS among other species, such as tilapini cichlids (Figure 2) [56] and balaenopterid baleen whales (Figure 4) [40]. In addition to our studies, ILS has been detected in the trichotomy of human–chimpanzee–gorillas, in which seven loci support a human–chimp grouping whereas one locus supports a human–gorilla grouping [27]. In addition, we have discovered signatures of rapid speciation among major groups of mammals through ILS detection using the SINE method, which will be discussed in the following sections.

### 3.2. Interordinal Relationships among Eutherian Mammals 

**Figure 6:**
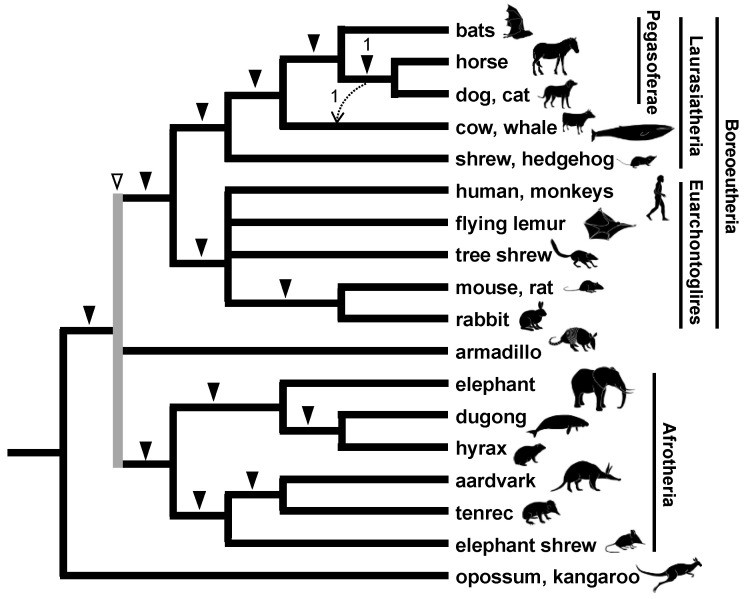
Phylogenetic relationship among mammalian orders as revealed by retroposon insertions. Only one locus denoted by a dashed arrow supports the monophyly of Cetartiodactyla, Perissodactyla, and Carnivora. The gray line, marked by an open arrowhead, indicates the trichotomic relationship between Boreoeutheria, Xenarthra, and Afrotheria.

The phylogenetic relationships among nearly 20 orders of eutherian mammals have attracted the attention of numerous researchers, including morphologists [58] and molecular phylogeneticists [79]. In 2001, a global interordinal phylogenetic tree was proposed based on multi-gene comparisons [80], but several relationships remained to be resolved. One of the unresolved issues was the relationships within Laurasiatheria, comprising bats, carnivorans, pangolins, perissodactyls, cetartiodactyls, and eulipotyphlans. The release of the draft genomes for humans and other mammals allowed us to incorporate genome information into the SINE method. To elucidate relationships among higher orders of mammals, we developed a new approach using genome information to explore the insertion loci [81]. First, in contrast to the original SINE method, a LINE family, namely LINE-1 (L1), was selected as a phylogenetic insertion marker because L1 is widely distributed among mammals whereas SINEs have a lineage-specific origin and expansion [82]. Since LINE is used as a marker, it should not be called a SINE method by definition, but conventionally, we include it in the SINE method. Second, L1 insertions in short introns were screened using databases for the human, mouse, dog, and cow genomes. Information for all insertion sites of transposable elements is available in public databases, such as the UCSC genome browser database. Third, oligonucleotide primers were designed to target conserved sequences of exons flanking L1 with subsequent PCR to identify orthologous loci. This approach is called inter-exon PCR and used the genomic DNAs of various mammals. This combination of bioinformatics and wet experiments identified 44 phylogenetically informative loci for L1 insertions, which allowed the construction of a phylogenetic tree for eutherian mammals (Figure 6) [17]. Another research group applied a similar approach for eutherian interordinal phylogenetics [83]. One of our unexpected findings was that four L1 insertions were detected exclusively in the bat, dog, cat, and horse genomes. By considering the close relationship between carnivorans and pangolins, we proposed the novel clade Pegasoferae comprising Chiroptera, Ferae (Carnivora + Pholidota), Pholidota, and Perissodactyla (Figure 6). Pegasoferae has one conflicting L1 insertion, suggesting that ILS occurred owing to the rapid radiation of the laurasiatherian lineages. Some subsequent phylogenetic studies using large collections of genes supported Pegasoferae [84], but many molecular studies failed to provide sufficient evidence for any of the possible topologies among laurasiatherians. A more in-depth retroposon analysis suggested network-like laurasiatherian relationships, including a possible hybridization scenario involving Perissodactyla, Carnivora, and Chiroptera, similar to Pegasoferae, but other scenarios are equally likely [85]. A recent study with more extensive retroposon insertions favored the closer relationship of Perissodactyla/Ferae to Cetartiodactyla [19], but still no single topology has been significantly supported. Therefore, the complex evolutionary history of laurasiatherians remains controversial in both phylogenomics and retrophylogenomics. In either case, it is evident from the retroposon studies that ILS caused by rapid radiation or ancestral introgression is likely to have occurred in the ancestral lineages. This highlights the phylogenetic characteristics of Laurasiatheria in the mammalian tree.

### 3.3. Rapid Divergence of the Three Major Eutherian Groups and an Association with the Continental Distribution

**Figure 7:**
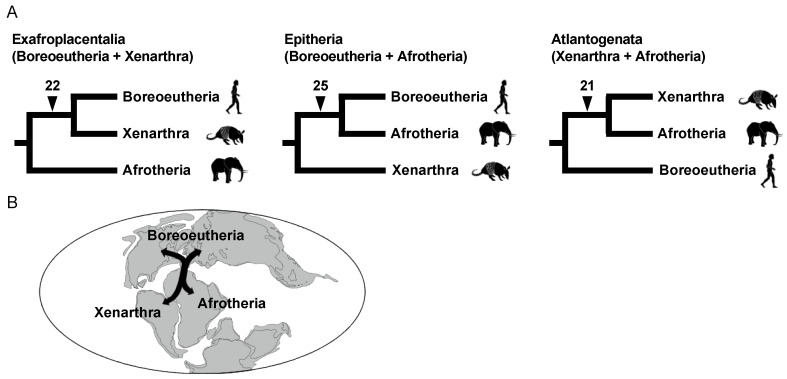
Radiation of the three major groups of Eutheria. (**A**) The three possible phylogenetic hypotheses are as follows: Exafroplacentalia (**left**), Epitheria (**middle**), and Atlantogenata (**right**). Each hypothesis is supported by the number of L1 insertions shown on each branch. (**B**) Possible scenario for eutherian radiation in concert with the separation of continents.

The root of the eutherian tree remains another critical issue of mammalian phylogenetics. Molecular phylogenetics and our retroposon insertion studies revealed that eutherian mammals can be classified into three major groups, namely Afrotheria, Xenarthra, and Boreoeutheria (or Boreotheria). However, the relationships among the three groups constituted a highly challenging issue—even based on the analyses of a large number of gene datasets [81]. This issue is noteworthy because species of these three groups inhabit different continents, i.e., Afrotheria, Xenarthra, and Boreoeutheria are considered to have originated in Africa, South America, and Laurasia (Eurasia + North America), respectively. Therefore, the divergence of the three lineages is considered to have coincided with continental drift on the geologic timescale [80]. It is believed that an ancient supercontinent was divided into the two supercontinents of Laurasia and Gondwana, the latter of which was further divided into Africa and South America based on the spatiotemporal distribution of magnetic stripes in the bedrock of the Atlantic Ocean floor [86]. Although some retroposon studies identified two L1 insertions that are shared between Boreoeutheria and Afrotheria and two other insertions shared between Xenarthra and Afrotheria, the evolutionary history of the split from the most recent common ancestor of Eutheria was poorly understood. We compared all L1 insertions available in data for the human, armadillo, and elephant genomes, as these species are representatives of Boreoeutheria, Xenarthra, and Afrotheria, respectively. As a result, 22, 25, and 21 L1 insertions were identified that support the basal Afrotheria, basal Xenarthra, and basal Boreoeutheria hypotheses, respectively (Figure 7A) [18]. This adequate number of retroposon loci that show conflict with each other is robust evidence for the occurrence of extensive ILS. Therefore, retroposon data revealed that the eutherian common ancestor diverged into three lineages almost simultaneously or over a short period. Another research group reached the same conclusion based on multiple incongruent retroposon insertion patterns [87].

Traditionally, the association between eutherian divergence and the separation of continents has been often discussed by referring to the spatiotemporal distribution of magnetic stripes [86]. However, magnetic stripes should not be used as the best reference because they do not directly reflect the timing of continental drift, and sea level changes were not considered. We investigated the latest on-land drilling data and found an indication that the continental divisions of Laurasia, Africa, and South America might have occurred over a shorter period than previously thought [18]. Therefore, we proposed that the near-simultaneous divergence of the three lineages might have been associated with the separation of continents (Figure 7B). This was the first report that the occurrence of rapid radiation was demonstrated by both an extensive number of retroposon insertions and geological analysis.

### 3.4. Advanced Retroposon Method with Next-Generation Sequencing Technology

**Figure 8:**
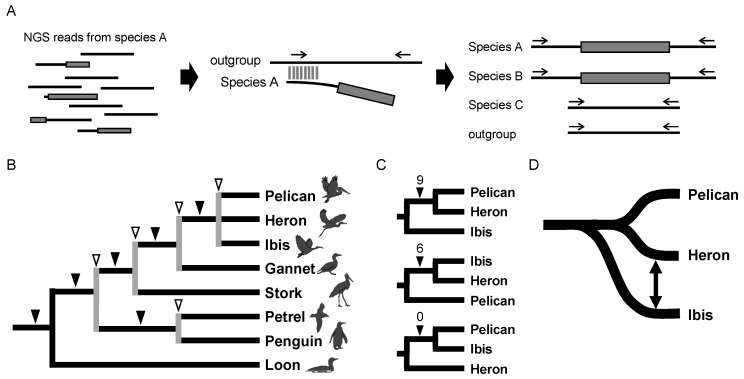
Waterbird phylogeny as revealed by results of the STRONG method. (**A**) The STRONG method. This method uses a collection of NGS reads (**left**) to search for orthologs from the genome database of an outgroup species (**middle**) and assess the presence/absence of retroposons by PCR (**right**). Gray shading denotes retroposons, and horizontal arrows above the solid lines indicate PCR primers. (**B**) Waterbird phylogeny as determined by retroposon insertion analysis. Gray lines indicated by open arrowheads denote nodes where ILS was detected based on data for two retroposon insertions. (**C**) Inconsistent retroposon insertions among the three phylogenetic hypotheses. Nine and six loci supported the close relationship between the pelican + heron clade and between the ibis + heron clade, respectively, whereas no insertions supported the pelican + ibis clade. (**D**) Proposed evolutionary scenario for the pelican, heron, and ibis lineages. Pelicans and herons are phylogenetically closely related, but gene flow might have occurred between the heron and ibis lineages.

The low cost of next-generation sequencing (NGS) technology allowed us to broaden the application of retroposon analysis. Although NGS is generally used for whole-genome assembly, a large number of sequence reads can be used to identify retroposons and their insertion loci if genome information is available for a closely related species. Based on this idea, we developed a method termed STRONG (Screening of Transposons Obtained by Next-Generation Sequencing) to directly screen insertion loci of retroposons from NGS reads (Figure 8A) [37]. The STRONG method enabled an analysis of one of the most debated issues in bird phylogenetics, namely the phylogenetic relationship among waterbirds, i.e., storks, pelicans, herons, ibises, penguins, etc. [36,88,89]. We first selected Illumina short fragments containing both CR1 and non-CR1 sequences, i.e., the insertion junctions. For each locus, the orthologous loci were identified in other bird genomes, such as chicken and zebra finch, and PCR primers were designed to target sequences conserved among species. A comparison of the 30 CR1 insertion patterns among waterbirds determined their complete phylogenetic relationship (Figure 8B) [37]. Furthermore, our bioinformatics approach detected an ancient cross-species introgression event based on incongruent retroposon analysis of heron, pelican, and ibis. The close relationships between heron + pelican and heron + ibis were supported by nine and six CR1 loci, respectively, whereas no locus was identified that supported the pelican + ibis grouping (Figure 8C). This result could not be explained by ILS because ILS should theoretically result in an equal number of inconsistent insertions, as was shown in the eutherian root [18]. Rather, asymmetric conflicts suggest the occurrence of ancestral hybridization between ancestral lineages [39,90]. In the case of the waterbirds, the number of loci supporting the heron + ibis and pelican + ibis hypotheses is not equivalent (6 vs. 0), even assuming heron + pelican is the species tree (Figure 8C). Therefore, the most likely rationale for the asymmetric pattern is that pelicans and herons are monophyletic among the three bird lineages and gene flow might have occurred between the ancestral lineages of herons and ibises (Figure 8D). Genome-scale phylogenetics suggested the close relationship between pelicans and herons with relatively weak support but did not raise the possibility of gene flow between the heron and ibis lineages that we proposed [91]. Thus, retroposon insertion, with a nearly homoplasy-free nature, is a reliable marker for detecting ILS that is indicative of ancient rapid radiation. Even though some incongruent retroposon loci were detected in the case of the waterbird phylogeny, the considerable increase in the number of loci as determined with the STRONG method allowed us to obtain statistically significant support for each clade. Furthermore, the biased numbers of incongruent insertions suggests gene flow caused by interspecies hybridization. In summary, use of NGS-based retroposon insertion analysis, such as our STRONG method, can greatly increase the number of loci for retroposon phylogenetics studies, enabling the evaluation of ancient interspecies hybridization events. The retroposon insertion analysis including the STRONG method will become more powerful by using the genomic data of a larger number of species. In birds, most of the families have at least one species for which genome assemblies are available, and a large-scale phylogenetic tree has been estimated using the dense-sampling genomics data [92]. Such data will be highly useful to clarify ancient occurrences of interspecies hybridization by using retroposon analysis in the future.

## 4. Conclusions

SINE (as well as other retroposon) insertions are ideal genetic markers for determining the genealogy of a specific locus, enabling the resolution of a number of difficult phylogenetic issues. The apparent problem of inconsistency in SINE insertion patterns among loci actually provided solid evidence for ILS that is attributable to rapid speciation. Even after the advent of NGS technology, the short-read data of HiSeq was, in practice, sometimes difficult to use for assembling genomic regions containing repetitive sequences, such as SINEs. However, this problem was solved with the development of long-read sequencing techniques, such as Nanopore and PacBio. A whole-genome sequence with unbiased coverage of many species would solve the problem of ascertainment bias. Whole-genome sequencing of multiple individuals within a species can also be applied to population genetics. Indeed, whole-genome sequence data can be used to optimize the efficiency of the SINE method to provide comprehensive inter- and intraspecies trees of life.
